# Androgen receptor-mediated apoptosis in bovine testicular induced pluripotent stem cells in response to phthalate esters

**DOI:** 10.1038/cddis.2013.420

**Published:** 2013-11-07

**Authors:** S-W Wang, S S-W Wang, D-C Wu, Y-C Lin, C-C Ku, C-C Wu, C-Y Chai, J-N Lee, E-M Tsai, C-LS Lin, R-C Yang, Y-C Ko, H-S Yu, C Huo, C-P Chuu, Y Murayama, Y Nakamura, S Hashimoto, K Matsushima, C Jin, R Eckner, C-S Lin, S Saito, K K Yokoyama

**Affiliations:** 1 Graduate Institute of Medicine, College of Medicine, Kaohsiung Medical University, Kaohsiung 807, Taiwan; 2 Department of Internal Medicine, College of Medicine, Kaohsiung Medical University Hospital, Kaohsiung 807, Taiwan; 3 Cancer Center, Kaohsiung Medical University Hospital, Kaohsiung 807, Taiwan; 4 School of Dentistry, Kaohsiung Medical University, Kaohsiung 807, Taiwan; 5 Graduate Institute of Clinical Medical Science, College of Medicine, China Medical University, Taichung 40402, Taiwan; 6 Institute of Cellular and System Medicines, National Health Research Institutes, Miaoli 35053, Taiwan; 7 College of Engineering, Nihon University, Koriyama, Fukushima 963-8642, Japan; 8 RIKEN BioResource Center, Tsukuba, Ibaraki 305-0074, Japan; 9 Department of Molecular Preventive Medicine, Graduate School of Medicine, The University of Tokyo, Tokyo 113-003, Japan; 10 Department of Environmental Medicine, NYU School of Medicine, Tuxedo, NY 10987, USA; 11 Department of Biochemistry and Molecular Biology, Rutgers New Jersey Medical School, Rutgers, The State University of New Jersey, Newark, NJ 07101, USA; 12 Saito Laboratory of Cell Technology, Yaita, Tochigi 329-1571, Japan

**Keywords:** environmental hormone, nuclear reprogramming, p53, testis cells, toxicity screening

## Abstract

The androgen receptor (AR) has a critical role in promoting androgen-dependent and -independent apoptosis in testicular cells. However, the molecular mechanisms that underlie the ligand-independent apoptosis, including the activity of AR in testicular stem cells, are not completely understood. In the present study, we generated induced pluripotent stem cells (iPSCs) from bovine testicular cells by electroporation of octamer-binding transcription factor 4 (*OCT4*). The cells were supplemented with leukemia inhibitory factor and bone morphogenetic protein 4, which maintained and stabilized the expression of stemness genes and pluripotency. The iPSCs were used to assess the apoptosis activity following exposure to phthalate esters, including di (2-ethyhexyl) phthalates, di (*n*-butyl) phthalate, and butyl benzyl phthalate. Phthalate esters significantly reduced the expression of AR in iPSCs and induced a higher ratio of BAX/BCL-2, thereby favoring apoptosis. Phthalate esters also increased the expression of cyclin-dependent kinase inhibitor 1 (p21^Cip1^) in a p53-dependent manner and enhanced the transcriptional activity of p53. The forced expression of AR and knockdown of p21^Cip1^ led to the rescue of the phthalate-mediated apoptosis. Overall, this study suggests that testicular iPSCs are a useful system for screening the toxicity of environmental disruptors and examining their effect on the maintenance of stemness and pluripotency, as well as for identifying the iPSC signaling pathway(s) that are deregulated by these chemicals.

Understanding the genotoxic or mutagenic risks of endocrine-disrupting chemicals (EDCs) is critical for the development of therapeutic agents against many diseases in humans and domestic animals.^1^ Thus, it is important to characterize the roles of genetic traits during development after exposure to EDCs. A promising, recently developed strategy is based on embryonic stem cells (ESCs) or induced pluripotent stem cells (iPSCs) and aims at supplying these cells or their derivatives to damaged human tissues to restore functionality. However, the effects on genetic traits and changes in the pluripotency and stemness of iPSCs during development caused by exposure to EDCs, especially environmental hormones such as phthalate derivatives, have not been characterized fully.

Phthalates are synthetic compounds, which are used widely as plasticizers, solvents, and additives in many consumer products. Several previous studies have reported that the main cellular targets of phthalates in the male reproductive organs are the Sertoli or Leydig cells of the testis.^2, 3, 4, 5^ The long-branched di-(2-ethylhexyl) phthalate (DEHP) and its metabolites have been shown to possess estrogen receptor *α* (ER*α*)-agonistic and ER*β*-antagonistic activities. By contrast, di (*n*-butyl) phthalate (DBP) and butyl benzyl phthalate (BBP) have ER*α*-agonistic activities and androgen receptor (AR)-antagonistic activities. DEHP and its metabolites can cause oxidative DNA damage to the testes by inducing apoptosis in testicular cells.^6^ Several selective ER modulators induce apoptosis in androgen-responsive prostate cancer cells via an androgen-independent pathway.^7^ A recent study demonstrated BBP-induced necrosis in human granulosa cells via its effects on the aryl hydrocarbon receptor.^8^ However, the effect of EDCs on apoptosis and necrosis in both ESCs and iPSCs remains unknown. The present study aimed to develop a method for screening drugs that might be used to treat the developmental diseases and regenerative disorders caused by EDCs, as well as to develop therapeutic agents that facilitate the maintenance of stemness and pluripotency.

The pluripotent ESC lines generated from domestic animals are useful for producing genetically modified livestock. The ESC cell lines hold great promise for the development of cell or organ therapies and drug screening and for use as human disease models. Many attempts have been made to establish ESCs in large domestic species, but teratoma formation displaying all three germ layers has only been confirmed in the goat.^9^

Pluripotent cells have been established from several embryonic and adult tissues using cell culture systems.^10^ For example, embryonic germ cells have been isolated from the primordial germ cells of midgestation embryos, while multipotent germline stem cells have been generated from explanted neonatal and adult mouse testicular cells, albeit at a very low efficiency.^11, 12, 13^ iPSCs have been generated by the addition of various combinations of transcription factors (octamer-binding transcription factor 4 (*OCT4*), *MYC*, *KLF4*, and *SOX2*).^14^

In this study, we characterized the stemness and pluripotency of bovine iPSCs generated by electroporation of *OCT4*. To understand the effects of environmental hormones such as phthalate derivatives on testicular iPSCs, we investigated the AR-mediated apoptosis of iPSCs. We also examined the global impact of phthalates on apoptosis induction and detected a novel molecular target for phthalates. We suggest that iPSCs could be useful for screening EDCs to determine their toxic effects during early development and on the pluripotency of stem cells in domestic animals. This screening method may provide a useful model for studying the effects of EDCs on human development.

## Results

### Stemness of iPSCs from bovine testicular cells

**Figure 1:**
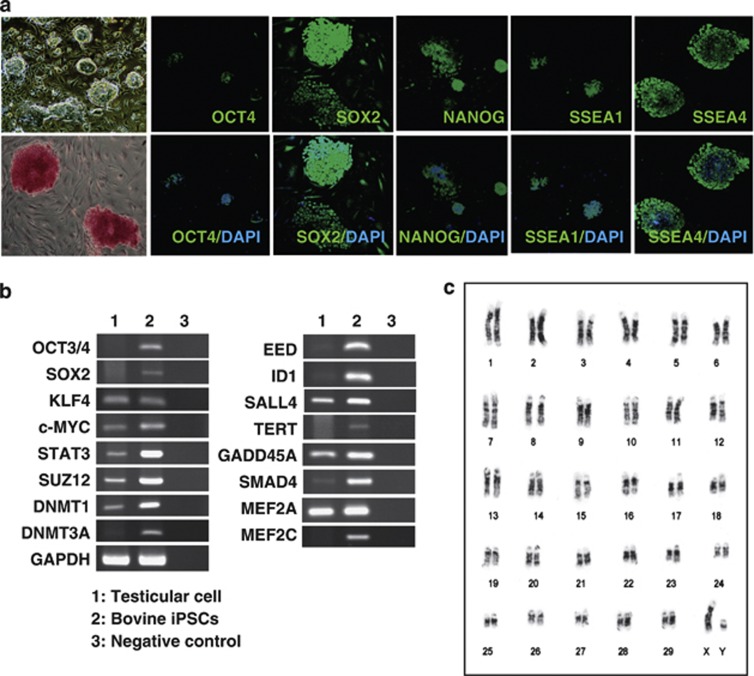
Generation of iPSCs from bovine testicular cells. (**a**) Typical morphology of bovine iPSC colonies generated using *OCT4* on day 25 after electroporation ( × 100 magnification; upper left panel). Alkaline phosphatase staining of bovine iPSCs (lower left panel), and immunocytochemical analysis of pluripotency and surface markers (OCT4, NANOG, SOX2, SSEA-1, and SSEA-4 indicated in green) in bovine iPSCs. Nuclei were stained with 4′,6-diamidino-2-phenylindole (indicated in blue) ( × 200 magnification). (**b**) Bovine iPSC gene expression. RT-PCR analysis of the transcripts of ‘stemness' genes (*OCT4*, *SOX2*, *MYC*, *KLF4*, *STAT3*, *SUZ12*, *DNMT1*, and *MEF2A*) in bovine testis cells and iPSCs. The primers used for RT-PCR are listed in Table 1. (**c**) G-banding karyotype analysis of the bovine iPSC cell line. Bovine iPSCs had the normal distribution of 60 chromosomes at passage 15, including the XY sex chromosomes

Compact, elliptical colonies were observed after three passages (15–21 days) of bovine testicular cells without a feeder cell layer. Several pluripotency markers, such as *KLF4*, *MYC*, *STAT3*, *DNMT1*, *SUZ12*, and *MEF2A*, were detected in the colonies, whereas other stemness markers were absent, including *OCT4*, *SOX2*, and *NANOG* (Figures 1a and b). We used electroporation to generate the bovine iPSCs, where the optimal conditions comprised 10 electrical pulses of 20 V at 50-ms intervals. Seventeen days after electroporation, we detected small, packed, domed colonies on the mitotic-inactivated mouse embryonic fibroblast (MEF) cells. These colonies comprised small, rapidly dividing cells with a high nuclear/cytoplasmic ratio and large nucleoli.^15^ The estimated reprogramming efficiency of our one-factor method was 0.3%, which is 20-fold higher than that of the one-factor approach used for reprogramming murine neural stem cells.^16^ The cells exhibited a strong alkaline phosphatase activity after we continued the culture for >4 weeks (Figure 1a). Immunofluorescence staining confirmed that the iPSCs induced by *OCT4* (1F-iPSCs) expressed stemness markers, such as OCT4, NANOG, SOX2, SSEA-1, and SSEA-4 (Figure 1a). These markers were more intense in the dense patches of cells. Reverse transcription-PCR (RT-PCR) analysis confirmed the expression of ESC markers in 1F-iPSCs, including *OCT4*, *SOX2*, *MYC*, *KLF4*, *MEF2a*, *SUZ12*, *STAT3*, and *DNMT1* (Figure 1b). A cytogenetic study based on G-banding demonstrated normal distributions of the 60 chromosomes in the iPSCs, including the XY sex chromosomes at passage 15 (Figure 1c).

### Pluripotency

**Figure 2:**
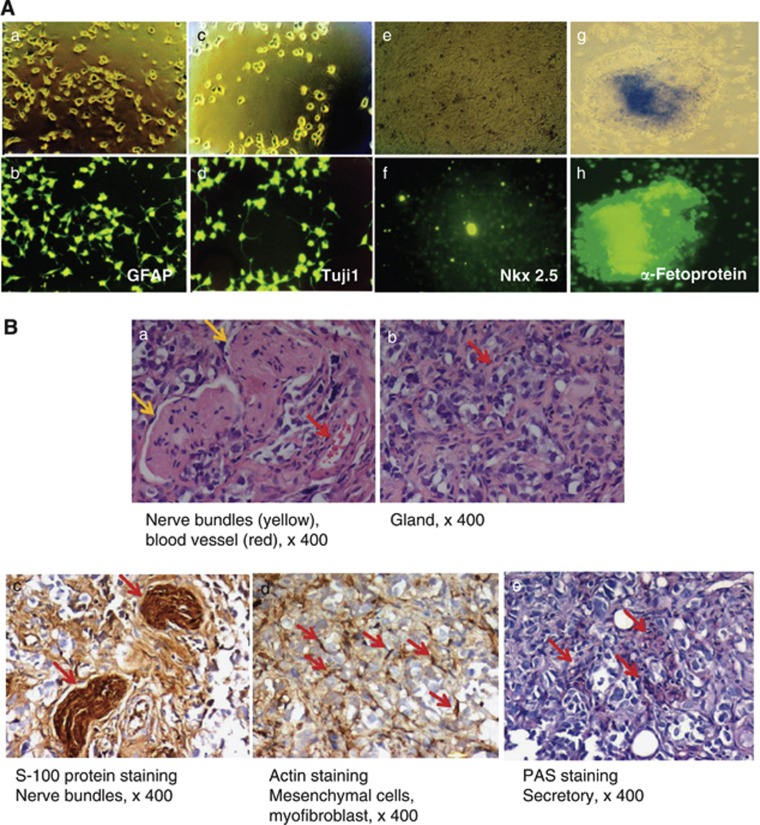
Pluripotency of bovine iPSCs. (**A**) *In vitro* differentiation of and marker expression by bovine iPSC-derived ectodermal, mesodermal, and endodermal precursor cells. Immunostaining with antibodies directed against the astrocyte-specific antigen GFAP (ectodermal differentiation), neuron-specific antigen Tuj1 (ectodermal differentiation), cardiomyocyte-specific antigen Nkx 2.5 (mesodermal differentiation), or *α*-fetoprotein (endodermal differentiation). (**B**) Teratoma formation 6–8 weeks after the transplantation of bovine iPSCs into SCID mice. Teratomas were sectioned and stained with hematoxylin and eosin. Immunohistochemical staining was performed using antibodies specific for S-100 (nerve bundles) and muscle-specific actin (mesenchymal cells and myofibroblasts) or PAS staining (secretory cells) ( × 400 magnification). In panel a, the red and yellow arrows indicate blood vessels and nerve bundles, respectively. In panel b, the red arrows indicate glands. S-100 staining indicates nerve bundles (panel c; red arrows), and muscle-specific actin staining indicates mesenchymal cells and myofibroblasts (panel d; red arrows). PAS staining indicates secretory cells (panel e; red arrows). The proliferation index of the whole teratoma was<3%

To confirm the developmental potential of the bovine 1F-iPSCs *in vitro*, the cell clumps were stimulated to differentiate into the three germ layers. Glial fibrillary acidic protein (GFAP)-positive astrocytes and anti-*β*-tubulin III (Tujl)-positive neurons, *α*-fetoprotein-positive endodermal cells, and Nkx 2.5-specific cardiomyocyte precursor cells were detected in most of the differentiated cell colonies (Figure 2A). To assess the pluripotency of the bovine 1F-iPSCs *in vivo*, we injected the cells into immunodeficient severe combined immunodeficiency (SCID) mice. The bovine iPSCs generated benign cystic teratomas with mature tissues expressing markers of the germ layers (Figure 2B). The differentiation into all three germ layers was confirmed by immunohistochemical staining for the neural marker S-100 and muscle actin and periodic acid-Schiff (PAS) staining, which are markers for the ectodermal, mesodermal, and endodermal lineages, respectively.

### Effects of phthalate esters

**Figure 3:**
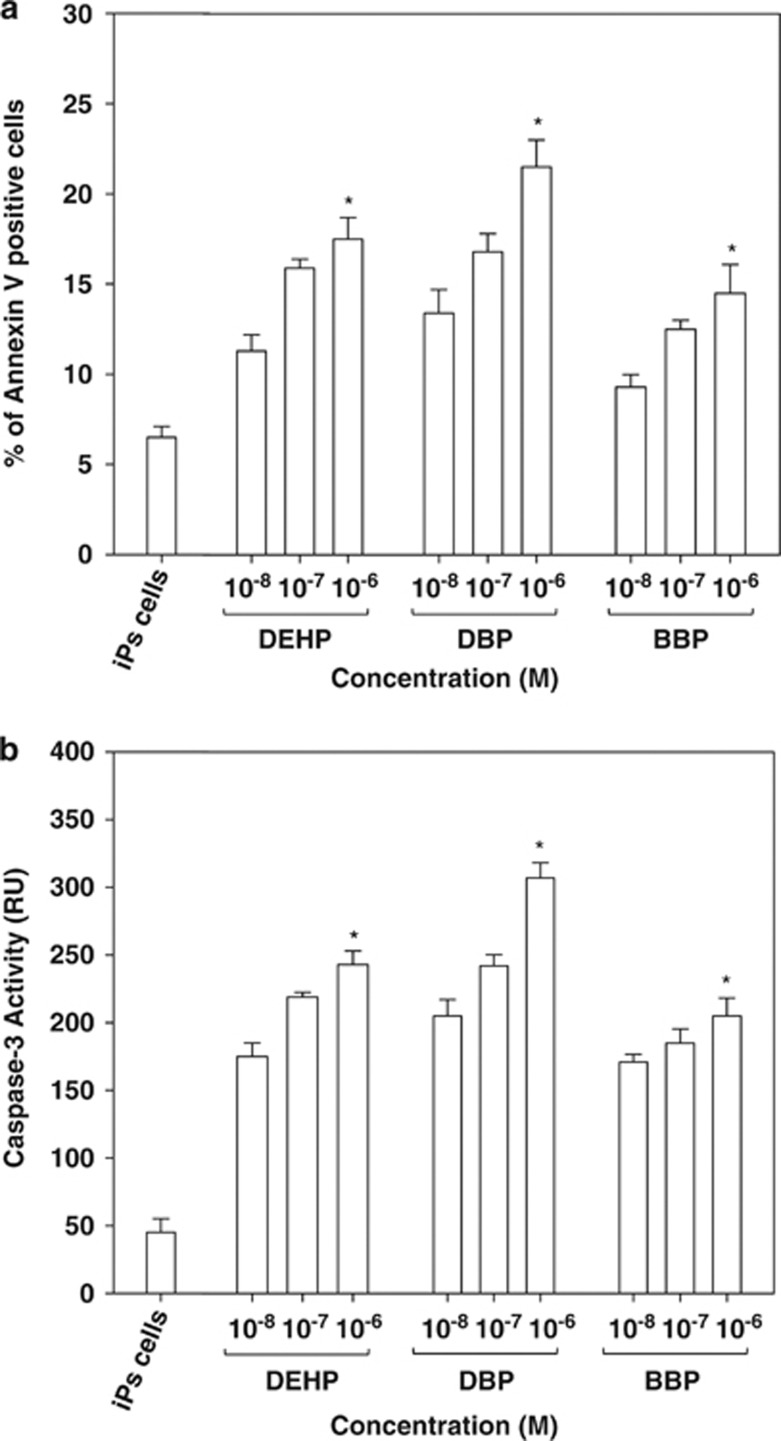
Apoptosis induced by phthalate derivatives in bovine iPSCs. (**a**) Fluorescein isothiocyanate-labeled annexin V staining followed by flow cytometry to identify apoptotic cells, as described in the Materials and Methods. DEHP, DBP, or BBP were added at doses of 10^−6^–10^−8^ M for 48 h, and their apoptotic activities were measured. (**b**) Caspase-3 activity was measured in iPSCs. DEHP, DBP, or BBP were added at doses of 10^−6^–10^−8^ M for 48 h, and their apoptotic activities were measured. Data were expressed as the means±S.D., and a *t*-test was used to compare them with the data obtained for DMSO-treated control iPSCs (*n*≥3, *P<0.05)

Next, we examined cytotoxicity, necrosis, and apoptosis in the bovine testicular cells and iPSCs generated from the same testicular cells following exposure to DEHP, DBP, and BBP. The three phthalates induced significant cytotoxicity in iPSCs compared with the original testicular cells, even at low concentrations (10^−6^ to 10^−8^ M; Supplementary Figure S1A). Interestingly, the phthalates induced a higher level of necrosis in the testicular cells compared with the iPSCs (Supplementary Figure S1B), whereas the phthalate esters elicited significant apoptotic activity in the iPSCs, which we evaluated using annexin V staining (about 2.2–3.3-fold; Figure 3a). This was also supported by the observations of a higher caspase 3 activity (about 4.5–6.8-fold; Figure 3b) and an increased sub-G1 cell population (about 5.2–8.4-fold; Supplementary Figure S1C) in the phthalate ester-treated iPSCs. These results suggest that the phthalate esters (DEHP, DBP, and BBP) induced apoptosis in bovine testicular cell-derived iPSCs.

### Screening specific antibodies for proteins from bovine iPSCs using a microwestern array (MWA)

To understand the signaling involved with apoptosis in testicular iPSCs exposed to phthalate esters, we used a MWA,^17^ which facilitated the high-throughput assessment of protein abundance after the electrophoretic separation of 96-well microarray cell lysates. We screened a series of antibodies to identify appropriate antibodies, which detected bovine and mouse proteins (Supplementary Figure S2A). To maintain the characteristic stemness of iPSCs, they had to be cultured with mitomycin C-treated MEF as feeder cells. Without the feeder cells, the stemness features were lost rapidly based on staining for alkaline phosphatase and SSEA 1 or 4 (data not shown). Thus, we had to examine samples from iPSCs with MEF and from MEF alone to compare the relative expression levels of apoptosis-related proteins (Supplementary Figure S2B). The results suggested that the protein levels of BAX and p21^Cip1^ (cycling-dependent kinase inhibitor 1) were increased in phthalate-treated iPSCs, which were normalized against the levels in MEF feeder cells.

### Increased BAX/BCL-2 ratio in phthalate ester-treated bovine testicular iPSCs

**Figure 4:**
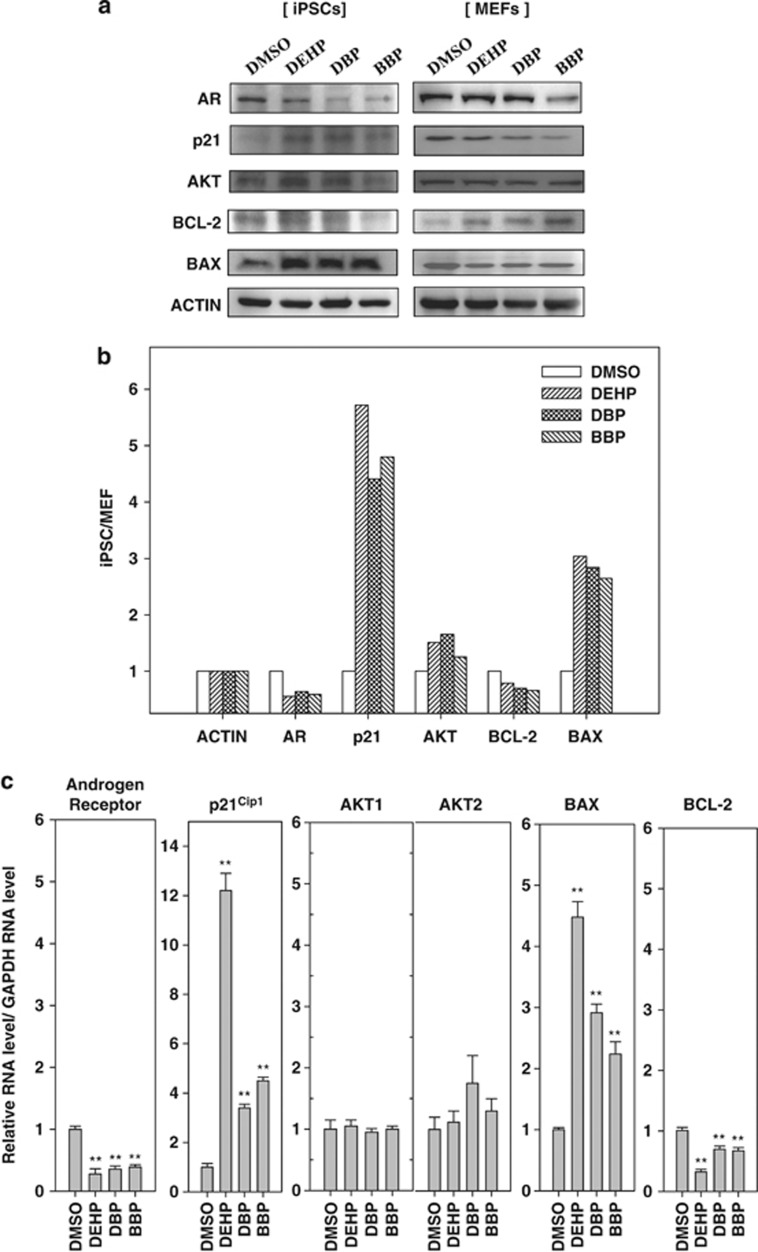
Effects of phthalates on apoptosis-related gene expression in bovine iPSCs and MEFs as feeder cells. (**a**) Western blotting analysis of the AR-mediated apoptosis-related proteins in cell lysates from iPSCs plus MEFs (left panels) and from MEFs alone (right panels). MEFs were treated with mitomycin C, cultured in the iPSC medium for 2 weeks, and treated with the phthalates indicated (0.1% DMSO-treated control, 10^−6^ M DEHP, 10^−6^ M DBP, and 10^−6^ M BBP) for 24 h, as described in the Materials and Methods, and then harvested. Proteins (30 *μ*g) were loaded into each lane, and each protein was detected using the antibodies indicated. (**b**) Relative expression values of the blotted proteins in iPSCs and MEF feeder cells. Blots were scanned and quantified using a LI-COR Odyssey near-infrared imaging system. *β*-Actin (control) was set as 1.0. Intensity of bands in western blotting was quantitated by GeneTools (Syngene) and Image Lab software (Bio-Rad). Relative intensities of each band image in iPSCs were calculated by normalization of corresponding band image of MEFs. (**c**) Relative mRNA expression levels of AR, p21^Cip1^, AKT1, AKT2, BAX, and BCL-2 in iPSCs were calculated. The expression level in the control (DMSO treated) was taken as 1.0. Cells were treated with phthalate derivatives (0.1% DMSO control, 10^−6^ M DEHP, 10^−6^ M DBP, and 10^−6^ M BBP). Real-time qPCR was performed using the bovine-specific primers, which were not cross-reacted with mouse, listed in Table 2. Data were expressed as the means±S.D., and a *t*-test was used to compare them with the results obtained for the DMSO-treated control iPSCs (*n*≥3, **P<0.01)

Next, we conducted traditional western blot analyses to verify the results obtained by MWA. Samples from iPSCs with MEF feeder cells and from MEF feeder cells alone were prepared as described above. We found that the expression level of the proapoptosis protein BAX was increased in iPSCs by treatment with DEHP, DBP, and BBP (about 2.6–3.0-fold, Figures 4a and b) after normalizing against the expression levels in MEF feeder cells. By contrast, the levels of antiapoptotic protein BCL-2 were low in iPSCs and MEF feeder cells (60–70% relative to the control of dimethyl sulfoxide (DMSO). After calculating the expression levels of BAX relative to BCL-2 based on *β*-actin expression, we found that there was a >4.0–4.3-fold increase in the BAX/BCL-2 ratio in iPSCs after exposure to phthalate esters compared with the control treatment using DMSO. Next, we examined the effects of phthalate esters on the mRNA levels of apoptosis-related genes by quantitative PCR (qPCR) using primers that specifically amplified bovine sequences but not mouse sequences. The expression levels of bovine-specific BAX mRNA were enhanced by 2.2–4.4-fold after the phthalate treatment compared with that using DMSO, whereas the expression levels of BCL-2 mRNA were decreased by 35–70% after treatment using phthalate esters compared with levels after iPSCs exposure to DMSO (Figure 4c). These results suggest that incubation with phthalate esters increases the BAXC/BCL-2 ratio and apoptosis in bovine testicular iPSCs.

### Regulation of AR, p21^Cip1^, and AKT expression by phthalates

Next, we examined the effects of phthalate derivatives on the expression of AR, p21^Cip1^, and AKT in iPSCs. Previous studies have found that AR has a role in apoptosis regulation in prostate cancer,^18, 19^ and both p21^Cip1^ and AKT are involved in AR-regulated apoptosis.^20, 21, 22, 23^ Using western blotting analysis, we found that treatments with the phthalate esters DEHP, DBP, and BBP reduced the AR expression level to 40, 55, and 45%, respectively, relative to the level of the DMSO-treated control (Figure 4b). The phthalates had no apparent effects on AR expression in mouse MEFs, whereas the AR levels were reduced in iPSCs. Thus, we conclude that the AR level was repressed by exposure to phthalate esters.

**Figure 5:**
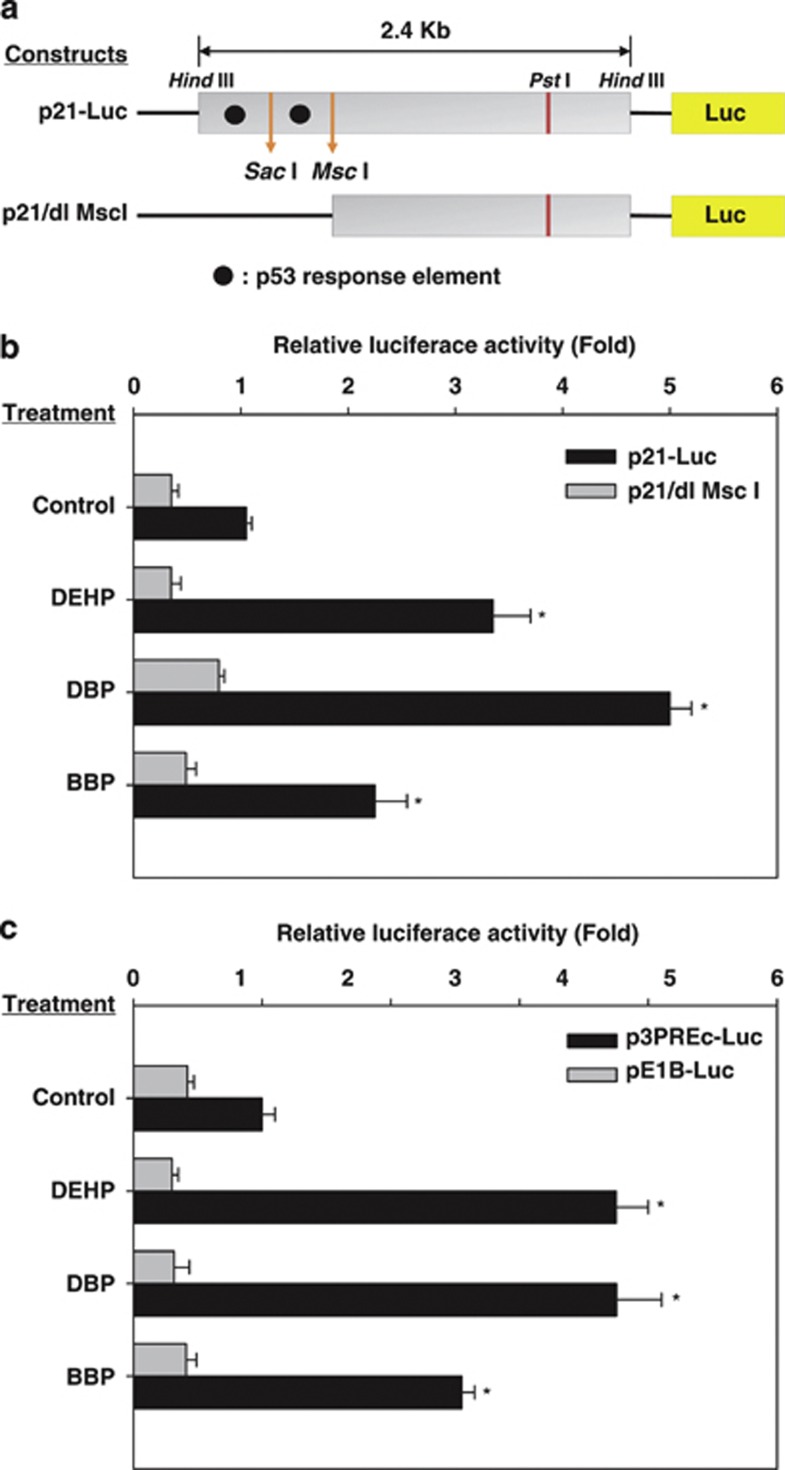
Activation of the p21^Cip1^ promoter by phthalate ester derivatives. (**a**) Schematic representation of p21^Cip1^ promoter reporter constructs. *p21-Luc*, wild-type p21^Cip1^ promoter; *p21/dl MscI*, mutant p21^Cip1^ promoter with deleted upstream and downstream p53 response elements. (**b**) Activation of the p21^Cip1^ promoter by phthalate ester derivatives (0.1% DMSO-treated control, 10^−6^ M DEHP, 10^−6^ M DBP, and 10^−6^ M BBP). Various *p21*^*Cip1*^ promoter reporter plasmids (400 ng) were transfected into iPSCs and mouse embryonic stem cells (MEFs). Luciferase activity in iPSCs was subtracted by the activity in respective MEFs. Relative luciferase activity was calculated as the ratio of the luciferase activity in iPSCs treated with phthalate esters relative to that in DMSO-treated control samples. Luciferase activity obtained by transfection of *p21-Luc* and treatment with DMSO (control) was set to 1.0. The values were expressed as means±S.D. and a *t*-test was used to compare them with the results obtained from DMSO-treated *p21-Luc*-transfected iPSCs (*n*≥3, *P<0.05). (**c**) Luciferase activity obtained by transfection with *p3PREc-Luc* (three copies of consensus p53 response elements) was calculated relative to that with *pE1B-Luc* (control reporter with minimal E1B TATA box). Luciferase activities in the respective MEFs were subtracted from those in the iPSCs. Cells were treated with phthalate derivatives (0.1% DMSO control, 10^−6^ M DEHP, 10^−6^ M DBP, and 10^−6^ M BBP). Treatment with DMSO (control) in pE1B-Luc was set to 1.0. Values were expressed as the mean±S.D., and a *t*-test was used to compare them with the results obtained from DMSO-treated p3PREc-Luc-transfected iPSCs (*n*≥3, *P<0.05)

By contrast, treatment using phthalate esters increased the p21^Cip1^ protein level in iPSCs but not in MEFs (4.0–5.7-fold increase; Figure 4b). The expression levels of p21^Cip1^ mRNA were increased in iPSCs treated with phthalates compared with DMSO-treated control iPSCs (Figure 4c). To confirm that the phthalate esters increased the expression of p21^Cip1^, we used a luciferase assay with a p21^Cip1^-promoter-luciferase construct (p21-Luc) and deletion mutants that lacked the two p53 response elements (p21/dl MscI) in the p21^Cip1^ promoter (Figure 5a).^24^ We transiently transfected the bovine iPSCs cells with these two p21-luciferase constructs. Treatment using the phthalate esters DEHP, DBP, and BBP increased the transcriptional reporter activity of the full-length p21-Luc by about 2.2–5.0-fold compared with that of the DMSO-treated control (Figure 5b). Loss of the two p53 binding sites, p21/dl MscI, reduced the luciferase activity to <20% compared with p21-Luc in the presence of phthalate esters. Moreover, p53 response elements-minimal promoter-luciferase constructs were also transiently transfected into iPSCs and the luciferase activity was measured (Figure 5c).^25^ The activity of p53 was increased significantly by treatment with phthalate, whereas the activity of the control vector pE1B-luc was not increased. These results demonstrated that treatment with phthalate esters increased the transactivation activity of p53.

### Role of AR and p21^Cip1^ in phthalate-mediated apoptosis

**Figure 6:**
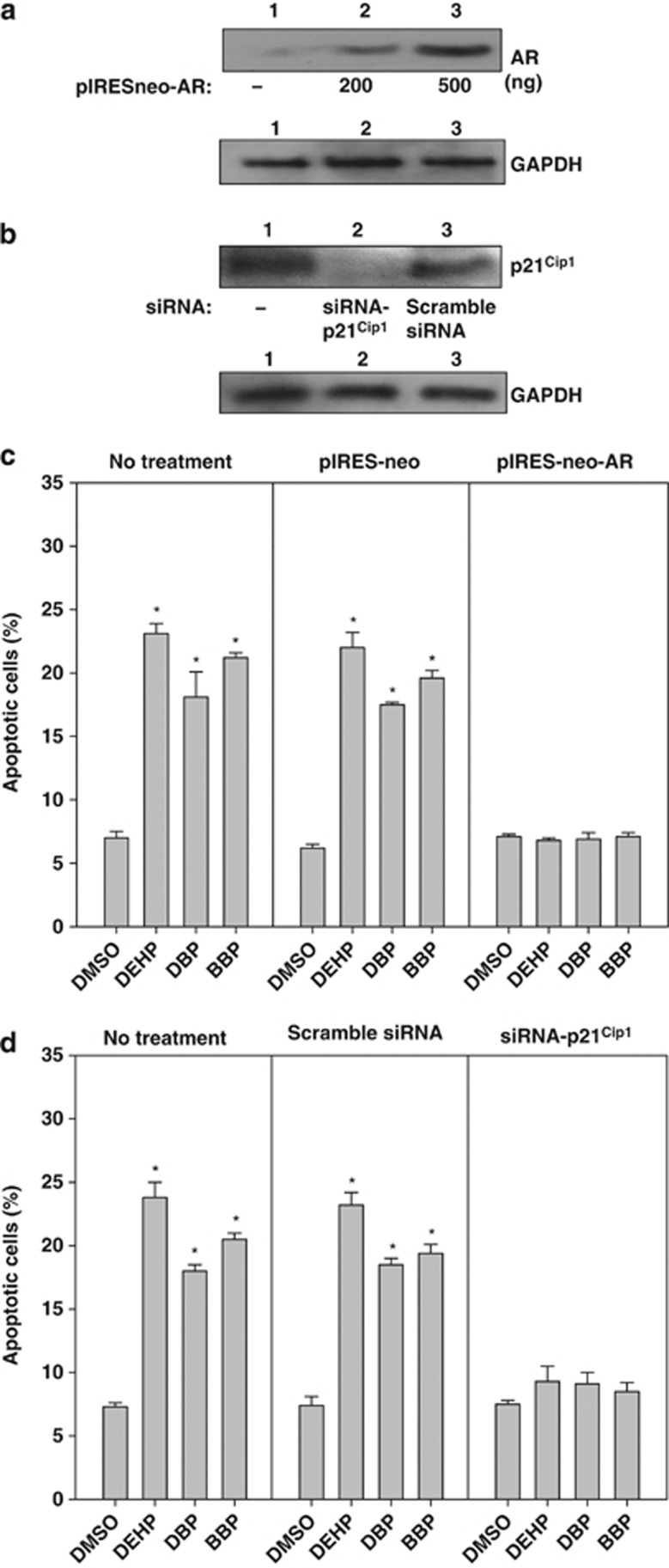
Effects of AR-forced expression and *p21*^*Cip1*^ siRNA knockdown expression on phthalate ester-induced apoptosis. (**a**) Protein expression of AR and (**b**) p21^Cip1^ in bovine iPSCs transfected with *pIRESneo-AR* and *p21*^*Cip1*^ siRNA, respectively. Four hundred nanograms of *pIRESneo-AR* or *p21*^*Cip1*^ siRNA and each control plasmid were introduced into bovine iPSCs, harvested at 24 h, and the respective proteins were identified by SDS-PAGE and western blotting analysis, as described in the Materials and Methods. The cells were cultured for 24 h, and the respective phthalate esters were added, followed by culture for another 24 h. (**c** and **d**) Apoptotic cells were quantified by staining with annexin V, as described in the Materials and Methods. (**c**) Effect of *pIRESneo-AR*. (**d**) Effect of *p21*^*Cip1*^ siRNA. Lane 1, 0.1% DMSO-treated control; lane 2, 10^−6^ M DEHP; lane 3, 10^−6^ M DBP; and lane 4, 10^−6^ M BBP. Data were expressed as the means±S.D., and a *t*-test was used to compare them with the results obtained with DMSO-treated control iPSCs (*n*≥3, *P<0.05)

To understand the link between phthalate-mediated AR repression and apoptosis induction, we introduced the *AR* expression vector into iPSCs and compared their sensitivity with phthalates (Figure 6). The forced expression of *AR* by *pIRESneo-AR* caused an approximately 5–6-fold increase in the expression of *AR*, but this was not the case with the control vector for *AR*, *pIRESneo* (Figure 6a). The apoptotic activity in *pIRESneo-AR*-transfected iPSCs induced by phthalates declined significantly to the control level, whereas the iPSCs transfected with the control vector for AR, *pIRES-neo*, did not exhibit this effect (Figure 6c). Similarly, the small interfering RNA (siRNA) against p21^Cip1^, but not scrambled siRNA, reduced the expression of p21^Cip^ (Figure 6b) and completely attenuated phthalate-induced apoptosis in bovine testicular iPSCs (Figure 6d). These results suggest that the apoptosis mediated by inactivation of *AR* and by the enhancement of p21^Cip1^ was induced by the exposure of bovine iPSCs to phthalate esters.

## Discussion

The results of this study have several important implications. First, the introduction of *OCT4* alone was sufficient to reprogram bovine testicular cells to generate iPSCs in the presence of leukemia inhibitory factor (LIF) and bone morphogenetic factor 4 (BMP4). Thus, the ectopic expression of *SOX2*, *KLF4*, and *MYC* is not required. Second, EDCs such as DEHP, DBP, and BBP induced more necrosis and less apoptosis in bovine testicular cells compared with bovine testicular iPSCs. Third, DHEP, DBP, and BBP induced significant apoptosis via the upregulation of BAX proapoptotic activity, AR downregulation, and the upregulation of *p21*^*Cip1*^.

ESCs are particularly sensitive to changes in the OCT4 dosage. For example, a 50% increase or decrease in the level of OCT4 causes their differentiation into cells that express endoderm and mesoderm or trophectoderm markers, respectively.^26^ Therefore OCT4 is a critical factor during nuclear reprogramming and cellular self-renewal. To the best of our knowledge, the generation of bovine iPSCs via transfection by *OCT4* alone has not been reported previously. It is widely accepted that OCT4 is essential for identifying pluripotent stem cells in mammalian embryos.^27, 28^ Contradictory studies have also shown that *OCT4* is not essential for the acquisition and maintenance of pluripotency during the generation of pig iPSCs^29, 30^ or for the self-renewal of mouse somatic stem cells.^31^ Therefore, the requirement for *OCT4* might be species-specific or cell-type specific, depending on the origin of the stem cells. In the present study, it was evident that *OCT4* alone was sufficient to induce pluripotency in bovine testis cells.

The expression of pluripotency markers, including *OCT4*, *NANOG*, *SOX2*, *STAT3*, *MYC*, *KLF4*, *TERT*, and *DNMT3A*, was maintained in the bovine iPSCs. The morphology of these iPSCs resembled that of mouse ESCs/iPSCs, rather than human ESCs/iPSCs. Mouse ESCs and iPSCs express SSEA-1 but not SSEA-4, whereas human ESCs and iPSCs express SSEA-4 but not SSEA-1.^32^ Pig iPSCs are also positive for SSEA-4 but not for SSEA-1 and exhibit a similar morphology to that of human ESCs/iPSCs.^29, 33^ Interestingly, bovine iPSCs express both SSEA-1 and SSEA-4, and SSEA-1 expression is observed in both equine and bovine embryonic stem-like cells, as we described previously.^15, 34, 35^ In addition to SSEA-1, we detected a strong signal for SSEA-4, which has not been reported previously in bovine ES-like cells.^15^ Therefore, our iPSCs are more similar to naive iPSCs than to iPSCs derived from fibroblasts.^36^ We found that bovine testis cells could be reprogrammed more easily than fibroblasts.

We used bovine iPSCs to examine the effects of EDCs, such as the phthalate derivatives DEHP, DBP, and BBP, on bovine testicular iPSCs. Phthalate ester derivatives increased necrosis in bovine testicular cells but induced apoptosis in bovine iPSCs (Figure 3 and Supplementary Figures S1B and S1C). Phthalate esters had a greater effect on apoptosis in iPSCs, which was correlated with the activation of BAX proapoptotic activity, downregulation of AR, and the upregulation of p21^Cip1^.

To understand phthalate ester-induced apoptosis in bovine iPSCs, we used several standard methods to isolate iPSCs from mouse MEFs as feeder cells, such as the immunobead method, fluorescence-activated cell sorting, the Matrigel culture method, and treatment with mild detaching enzyme. However, none of these methods obtained the pure and intact iPSCs. Thus, we used two methods to overcome this problem; (i) we designed bovine-specific qPCR primers to differentiate the gene expression of bovine iPSCs from that of mouse MEFs as feeder cells, and (ii) we compared the relative expression levels of apoptosis-related proteins in iPSCs with MEF feeder cells and in MEF feeder cells alone. We identified appropriate antibodies using MWA.^17^ This approach is very useful for the high-throughput assessment of protein-expression levels if only limited sample volumes are available. The level of BAX expression relative to BCL-2 proteins were higher in phthalate-treated iPSCs compared with the DMSO-treated control (4.0–4.3-fold for proteins; 3.1–14.6-fold for mRNAs), which demonstrated that the apoptosis-related protein levels were affected by the exposure of cells to phthalate esters (Figure 4).

The proapoptotic BCL-2 family protein BAX has a critical role in the intrinsic apoptotic pathway.^37^ Overexpression of BAX alone is sufficient to induce apoptosis^38^ and BAX also mediates the apoptotic signal from many death stimuli, including ultraviolet irradiation and ceramide.^37^

How do phthalate esters promote apoptosis? We found that the treatment of iPSCs with phthalate esters activated the transcriptional activity of p53 (Figure 5c), which is known to upregulate BAX and p21^Cip1^. Indeed, we found that the expression levels of BAX and p21^Cip1^ were increased by exposure to phthalate esters (Figure 4). The enhanced expression and activity levels of p53 by phthalate ester derivatives has also been reported in mouse osteoblast^39^ and contributed partly to phthalate-mediated osteoblast apoptosis. Our data suggest that p53 activation may be involved with the phthalate ester-induced apoptosis of bovine testicular iPSCs. Moreover, we found that phthalate-mediated apoptosis was regulated by p21^Cip1^, because knockdown using a siRNA against p21^Cip1^ caused a reduction in apoptosis in response to phthalate esters (Figure 6). A role for the increased expression of p21^Cip1^ during the induction of apoptosis was also suggested in glioma and ovarian carcinoma treated by cisplatin, in hepatocytes by bile acid, in colon cancer by C6 ceramide, and in differentiating granulocytes induced by granulocyte colony-stimulating factor.^40^ In beta cells, at least, p21^Cip1^ upregulation activated the intrinsic apoptotic pathway via BAX expression.^41^

However, the role of p21^Cip1^ in apoptosis may differ depending on the cell context. Several studies have suggested that p21^Cip1^ is an antiapoptotic factor. These studies showed that DNA-damaging agents, oxidative stress, TGF-*β*, tumor necrosis factor-*α*, and other inducers caused p21^Cip1^ expression, irrespective of p53-dependent or -independent apoptosis.^20, 21^

At present, there is no explanation for this apparent inconsistency, but phthalates clearly induced the increased expression of p21^Cip1^ in bovine iPSCs, which resulted in apoptosis.^42^

AR has a prosurvival function in androgen-dependent prostate cancer cells, which are susceptible to apoptosis without AR expression. In the present study, AR expression was reduced in bovine testicular iPSCs after exposure to phthalate esters (Figure 4), which increased apoptosis by 2–3-fold compared with the treatments that lacked phthalate esters (Figure 3). To clarify the role of AR in phthalate-mediated apoptosis in bovine testicular iPSCs, we introduced an AR expression vector and found that it could rescue phthalate ester-mediated apoptosis. Therefore, our data suggest that AR expression is critical for the survival of bovine testicular iPSCs in response to phthalate esters.

At present, it is unclear how phthalate esters repress AR expression. Our preliminary data suggest that Wnt-*β*-catenin signaling may be critical, because overexpression of Frizzled 7 rescued the phthalate-mediated repression of AR mRNA expression and its promoter activity (by 6-fold and 3-fold, respectively; Supplementary Figures S3A and S3B). Frizzled 7 also rescued phthalate-induced apoptosis (Supplementary Figure S3C), which suggests a functional role for Wnt-*β*-catenin/AR signaling in bovine testicular iPSCs in response to phthalate esters. However, the precise mechanism needs to be elucidated by further experiments.

In summary, we generated iPSCs from bovine testicular cells by electroporation of *OCT4*. Exposure of these iPSCs to DEHP, DBP, and BBP repressed the expression of AR and increased expression of p21^Cip1^, both of which committed the iPSCs to apoptosis. Thus, these testicular iPSCs are useful for screening drugs that may protect from EDC-mediated cytotoxicity by maintaining the stemness and pluripotency of stem cells.

## Materials and Methods

### Reagents and plasmids

DBP, BBP, and DEHP were purchased from Sigma-Aldrich (St. Louis, MO, USA). The caspase 3 assay kit was obtained from Promega (Madison, WI, USA). Trypan blue stain solution (0.5%) was supplied by Nacalai Tesque Inc. (Kyoto, Japan). Biotin-conjugated 16–2′-deoxyuridine-5′triphosphate, proteinase K, and the blocking reagent were obtained from Roche Diagnostics (Mannheim, Germany). pCMV-Flag-hOCT3/4 (RDB6598) was obtained from the RIKEN DNA Bank (Tsukuba, Japan) and the pEGFP plasmid was generated as described previously.^15^ The plasmids, pIRESneo-AR, WT-ARE-luciferase, mutARE-luciferase, and pGK-CAS-FZD7, were kind gifts from Dr. Ben H. Park (The Sidney Kimmel Comprehensive Cancer Center at Johns Hopkins, Baltimore, MD, USA), Dr. Patrice J. Morin (National Institute on Aging, National Institutes of Health, Baltimore, MD, USA), and Dr. Karl Willert (University of California, San Diego, CA, USA), respectively. The siRNA construct against *p21*^*Cip1*^ was obtained from Invitrogen (Carlsbad, CA, USA).

### Culture of bovine testicular cells

The testicular tissues from a bull calf were cut into 1–3 mm^3^ pieces and isolated by enzymatic digestion using 0.25% trypsin-EDTA (Gibco, Grand Island, NY, USA) for 10 min, followed by culture in the iPSC medium without BMP4 (Dulbecco's modified Eagle's medium (DMEM; Gibco) containing 10 ng/ml human inhibitor factor (LIF) (Sigma-Aldrich) and supplemented with 10% fetal bovine serum (FBS), and antimycotics-antibiotics (AM-AB; Gibco)). After 2–3 passages, compact colonies were picked and split into other dishes at a 1 : 3 ratio in the same medium.

### Generation of iPSCs

The dissociated testicular cells (5 × 10^5^) were used for transfection with the *OCT4* gene as described elsewhere,^43^ where 10 direct-current electrical pulses at a 20 V intensity were applied at an interval of 50 ms. Cells in 2-mm cuvettes containing 200 *μ*l of DMEM and 10 *μ*g of plasmid DNA were treated in an electroporator (CUY21Vitro-EX; BEX, Tokyo, Japan). The cells were then cultured and selected with G418 (100 *μ*g/ml). Two days after selection, the cells were replated onto mitomycin-C-treated MEFs using the standard iPSC-medium supplemented with BMP4 (5 ng/ml; Sigma-Aldrich). The transfected cells were grown in the same medium until iPSCs were detected on day 17. The iPSC colonies were then picked up manually and replated onto a new feeder layer (first passage). The bovine iPSCs were then subcultured with trypsin-EDTA treatment, and the medium was replaced every 2 days. The bovine iPSCs (2 × 10^5^) were incubated for 24 or 48 h in the presence of the phthalate esters, DEHP, DBP, or BBP (Sigma-Aldrich), at the indicated doses and then harvested.

### Stemness assay and karyotyping

The alkaline phosphatase activity and immunostaining were determined as described previously.^43^ The antibodies were directed against OCT4 (sx-5279; Santa Cruz Biotechnology, Santa Cruz, CA, USA), NANOG (AF1997; R&D Systems, Minneapolis, MN, USA), SOX2 (AB5603; Millipore, Billerica, MA, USA), SSEA-1 (MAB4301; Millipore), and SSEA-4 (MAB4304; Millipore), and the fluorescently labeled secondary antibodies A11034 and A11029 were obtained from Invitrogen. Nuclei were detected with 0.5 *μ*g/ml 4′,6-diamidino-2-phenylindole (DAPI, D3571; Invitrogen) for 1 h. Metaphase mitotic chromosomes were prepared using a conventional air-drying technique. GTG (G-banding) staining was performed as described elsewhere.^44^

### Cell viability, apoptosis, and necrosis

The number of viable cells was determined using a LIVE/DEAD Viability/Cytotoxicity Assay Kit (L-3224; Life Technologies, Grand Island, NY, USA) according to the manufacturer's protocol. To differentiate apoptosis from cell necrosis, cells were identified by the flow cytometric analysis of cells stained with fluorescein isothiocyanate (FITC)-labeled annexin V to identify apoptotic cells and propidium iodide was used to label permeable cells (FITC Annexin V Apoptosis Detection Kit II; BD Biosciences, San Jose, CA, USA). The percentages of necrotic cells were determined using an Apoptotic/Necrotic Cells Detection Kit (PK-CA 707-30017; PromoCell GmbH, Heidelberg, Germany). The caspase-3 assay was also conducted as described elsewhere.^45^

### Cell cycle analysis

Cells were fixed with 70% ethanol and stained with PI (50 *μ*g/ml) in the presence of RNAase A (100 U/ml). PI-stained cells were detected with the FL-2 photomultiplier of a FACScalibur flow cytometer (BD Biosciences). The proportions of cells in the different cell cycle phases were determined. The fraction of apoptotic cells was quantified based on the analysis of the sub-G1 peak (sub-diploid cells).^46^ The sub-G1 fraction was determined by FACS analysis.

### Western blotting analysis

Cells were lysed in sodium dodecyl sulfate (SDS) lysis buffer (240 mM/l Tris-acetate, 1% SDS, 1% glycerol, 5 mM/l EDTA, pH 8.0) with dithiothreitol, protease inhibitors, and a cocktail of phosphatase inhibitors. The expression levels of proteins were examined using the following antibodies; AR (N-20: sc-816; Santa Cruz Biotechnology), p21 (C-19: sc-397; Santa Cruz Biotechnology), and AKT (Epitomics, Burlingame, CA, USA), *β*-actin, BAX (2772), and Bcl-2 (2870) (the latter three were obtained from Cell Signaling Technology, Beverly, MA, USA). Anti-rabbit and anti-mouse immunoglobulin (IgG) secondary antibodies were supplied by Invitrogen. The intensities of the bands produced by western blotting were quantified using GeneTools (Syngene, Cambridge, UK) and Image Lab software (Bio-Rad, Hercules, CA, USA). The relative intensities of each band image from the iPSCs and MEFs were calculated separately by normalizing against *β*-Actin. Each band image from the iPSCs was then divided by the values in the corresponding band images from the MEFs.

### MWAs

The cells were lysed at the time points indicated, and MWAs were conducted to measure the protein expression levels and changes, as described previously.^17^ The blots were scanned and quantified using a LI-COR Odyssey near-infrared imaging system. *β*-Actin and glyceraldehyde-3-phosphate dehydrogenase (Millipore) were used as the loading controls. The intensities of the bands produced by western blotting were quantified using GeneTools (Syngene) and Image Lab software (Bio-Rad). The relative intensities of each band image from the iPSCs were calculated by normalizing against the corresponding band images from MEFs as 1.0.

### RNA extraction, RT-PCR, and qPCR

RNA was extracted from cells in the presence of the indicated dose of DEHP, DBP, BBP, and DMSO, as described elsewhere.^46, 47, 48^ RNA was purified using an RNeasy Mini kit (2074104; Qiagen, Hilden, Germany), and RT was performed using Superscript III reverse transcriptase (18080-093; Invitrogen) and primers (Table 1). PCR was performed using GoTaq Green Master Mix (M7122; Promega). To avoid contamination by feeder cells, we selected primer pairs that did not amplify mouse transcripts. Real-time quantitative RT-PCR (qPCR) was performed using a PRISM 7700 system as described elsewhere (Amersham Biosystems, Foster City, CA, USA).^46, 47, 48^ We designed the primers with the public-domain Primer 3 program in GENETYX-Mac Ver. 14 (Hitachi Software, Tokyo, Japan). The respective pairs of primers are listed in Table 2.

### Transfection and luciferase assay

pIRESneo-AR, pIREneo, p21-Luc, p21/dlMscI, p3PREc-Luc, and pE1B-Luc were transfected into bovine iPSCs and MEFs at 400 ng with the total DNA per well of a 24-well plate (5 × 10^4^ cells/well) using 2 *μ*l of lipofectamine-2000 reagent (Invitrogen) and cultured in the presence of the indicated amount of phthalate ester. The luciferase activity was then measured using an assay kit system (Dual-Glo; Promega), as described elsewhere.^25, 46, 47, 48^ Twenty-four hours after phthalate treatment, the luciferase activity was measured using a commercial luciferase assay system (Dual-Glo). The relative luciferase activity was expressed as the ratio of the luciferase activities in iPSCs and MEFs. The control activity levels are obtained from cells treated with DMSO.

### *In vitro* differentiation analysis

Bovine iPSCs were harvested using trypsin, and the large clumps of cells (around 100 cells) isolated after centrifugation were plated in differentiation medium in six-well dishes. To induce ectodermal (neuronal) differentiation, the cells were cultured in medium (DMEM, 10 ng/ml basic fibroblast growth factor, 10 ng/ml EGF, 10 ng/ml platelet-derived growth factor, and 1% AM-AB) for 7 days, followed by culture in growth medium (DMEM, 10% FBS, and 1% AM-AB) for 7–14 days. To induce mesodermal (cardiomyocyte) differentiation, the cell colonies were placed in suspension culture in differentiation medium (DMEM, 10% FBS, 100 *μ*M ascorbic acid, and 1% AM-AB) for 10 days. The cell clumps were placed in gelatin-coated dishes in the same medium, and the adherent cardiomyocytes were observed at 7 days after replating. To induce endodermal differentiation, the cells were differentiated in medium (DMEM, 100 ng/ml activin-A, and 1% AM-AB) for 7 days and then transferred to growth medium (DMEM, 10% FBS and 1% AM-AB), where they were allowed to differentiate for 7 days. The following antibodies were used: mouse anti-astrocyte-specific GFAP antibody (Sigma-Aldrich), mouse neuron-specific Tuj1 antibody (Sigma-Aldrich), mouse anti-cardiomyocyte-specific anti-human Nkx 2.5 antibody (CosmoBio, Tokyo, Japan), and mouse anti-endoderm-specific anti-human *α*-fetoprotein protein (CosmoBio). FITC-conjugated rabbit secondary antibody against mouse IgG (Sigma-Aldrich) was used for immunostaining.

### Teratoma formation assay

Bovine iPSCs (2 × 10^6^) in DMEM plus 10% FBS were injected under the kidney capsule of SCID mice using a 27-G needle. Six-to-8 weeks after injection, the tumors were dissected surgically, fixed with 4% formaldehyde, embedded in paraffin, and 4-*μ*m sections were cut and stained with hematoxylin and eosin. The following antibodies were used: rabbit anti-human muscle-specific actin (M0635; Dako, Glostrup, Denmark), rabbit anti-human S-100 (N1573; Dako), rabbit anti-human epithelial membrane antigen (M0613; Dako), and rabbit anti-human cytokeratin (M3515; Dako). PAS staining was performed according to the manufacturer's instructions (NovaUltra Special Staining Kits; Woodstock, MD, USA).

### Statistical analysis

All of the data were expressed as the mean±S.D. The differences between the untreated control and phthalate-exposed groups were analyzed using a one-way analysis of variance, followed by Dunnett's test. Differences were considered statistically significant if *P*<0.05.

## Figures and Tables

**Table 1:** Nucleotide sequences of the primers used for stemness-related genes and the expected sizes of the DNA amplicons

	**Gene**	**5′→3′**	**Size of amplified DNA (bp)**
1	OCT3/4-F	CCCTGAGGAGTCCCAGGACAT	356
	OCT3/4-R	GCAGGAACATGCTCTCCAGGTT	
2	SOX2-F	CTACAGCATGATGCAGGACCAGCT	381
	SOX2-R	TGCTGGGACATGTGAAGTCTGCTG	
3	GKLF4-F	GTTCGTGTTGAAGGCGTCGCTG	173
	GKLF4-R	TGCACGAGGAGACAGCCTCCT	
4	c-MYC-F	CCAAGCTCGTCTCGGAGAAGC	334
	c-MYC-R	TCAGAGTCGCTACTGGTCGTGG	
5	SALL4-F	CATAGACAAGGCCACCACCGACC	276
	SALL4-R	ATGTGCATGCGGATGTGCTGCT	
6	ID1-F	ACGACATGAACGGCTGCTACTC	142
	ID1-R	TGGGATTCCGAGTTGAGCTCCAA	
7	EED-F	ATAGCAATACAAGCCATCCCCTGC	223
	EED-R	AATATTGCCACCAGAGTGTCCGTC	
8	SUZ12-F	GCAGTTCACTCTTCGTTGGACAGG	449
	SUZ12-R	CCTGAGGATTTCCTGCATAGGAGC	
9	STAT3-F	GTCTAACAATGGCAGCCTCTCAGC	405
	STAT3-R	AAGAGTTTCTCCGCCAGCGTC	
10	GADD45A-F	CTTTGGAGGAATTCTCGGCTGGAG	252
	GADD45A-R	CATTCTCACAGCAGAATGCCTGG	
11	SMAD4-F	TTCATGACTTTGAGGGACAGCCA	438
	SMAD4-R	GCTCATTGTGAACTGGTGGCCAG	
12	DNMT1-F	CGGTGTTCACAAAGGACTGCAACG	359
	DNMT1-R	GTACTGACCAGCCTGCAGCAC	
13	DNMT3A-F	TGCAAGAACTGCTTCCTGGAATGC	398
	DNMT3A-R	ACCAGAAGCCCTGTAGCAATTCC	
14	TERT-F	CCTACGTGGTGGAGCTGCTCAG	155
	TERT-R	TGACAGTTCTCGAAGCCGCAC	
15	MEF2A-F	ATGCCTCCACTGAATACCCAAAGG	217
	MEF2A-R	ACACCTGTCCCAGAGACAGCAT	
16	MEF2C-F	GGTATGGCAATCCCCGAAACTCAC	408
	MEF2C-R	GCCAGCCAGTTACTGACCCAAGAT	

**Table 2:** Nucleotide sequences of the primers used for quantitative PCR (qPCR)

	**Gene**	**5′→3′**
1	Androgen receptor-F	CAGTGGATGGGCTGAAAAAT
	Androgen receptor-R	AGGAGCTTGGTGAGCTGGTA
2	p21^Cip1^-F	ATGGGTCTGGGAGATGTGAG
	p21^Cip1^-R	CATATGGGAGCCAGGAGAAA
3	AKT1-F	GGTGAAGGAGAAGGCCACAG
	AKT1-R	TACTTCAGGGCCGTCAGGG
4	AKT2-F	TTGGCTATAAGGAGCGGCCT
	AKT2-R	TCTCGTCTGGGGAGTCAACA
5	BAX-F	ATGGACGGGTCCGGGGAGCAA
	BAX-R	TCAGCCCATCTTCTTCCAGAT
6	BCL-2-F	GCATCGTGGCCTTCTTTGAGT
	BCL-2-R	TGAGCAGTGCCTTCAGAGACAG
7	GAPDH-F	GGGTCATCATCTCTGCACCT
	GAPDH-R	GGTCATAAGTCCCTCCACGA

## Abbreviations

AR: androgen receptor
BBP: butyl benzyl phthalate
DBP: di (*n*-butyl) phthalate
DEHP: di-(2-ethylhexyl) phthalate
DMSO: dimethyl sulfoxide
EDC: endocrine-disrupting chemical
iPSC: induced pluripotent stem cell
MEF: mouse embryonic fibroblast
MWA: microwestern array
OCT4: octamer-binding transcription factor 4
p21^Cip1^: cycling-dependent kinase inhibitor 1
qPCR: quantitative PCR
RT-PCR: reverse transcription-PCR
