# Supplementary material for: Androgen receptor-mediated apoptosis in bovine testicular induced pluripotent stem cells in response to phthalate esters
Source: Cell Death Dis. 2013 Nov 7;4(11):e907–. doi: 10.1038/cddis.2013.420 (PMC3847308; doi:10.1038/cddis.2013.420)
Supplement: Supplementary Figures [file cddis2013420x1.pdf]

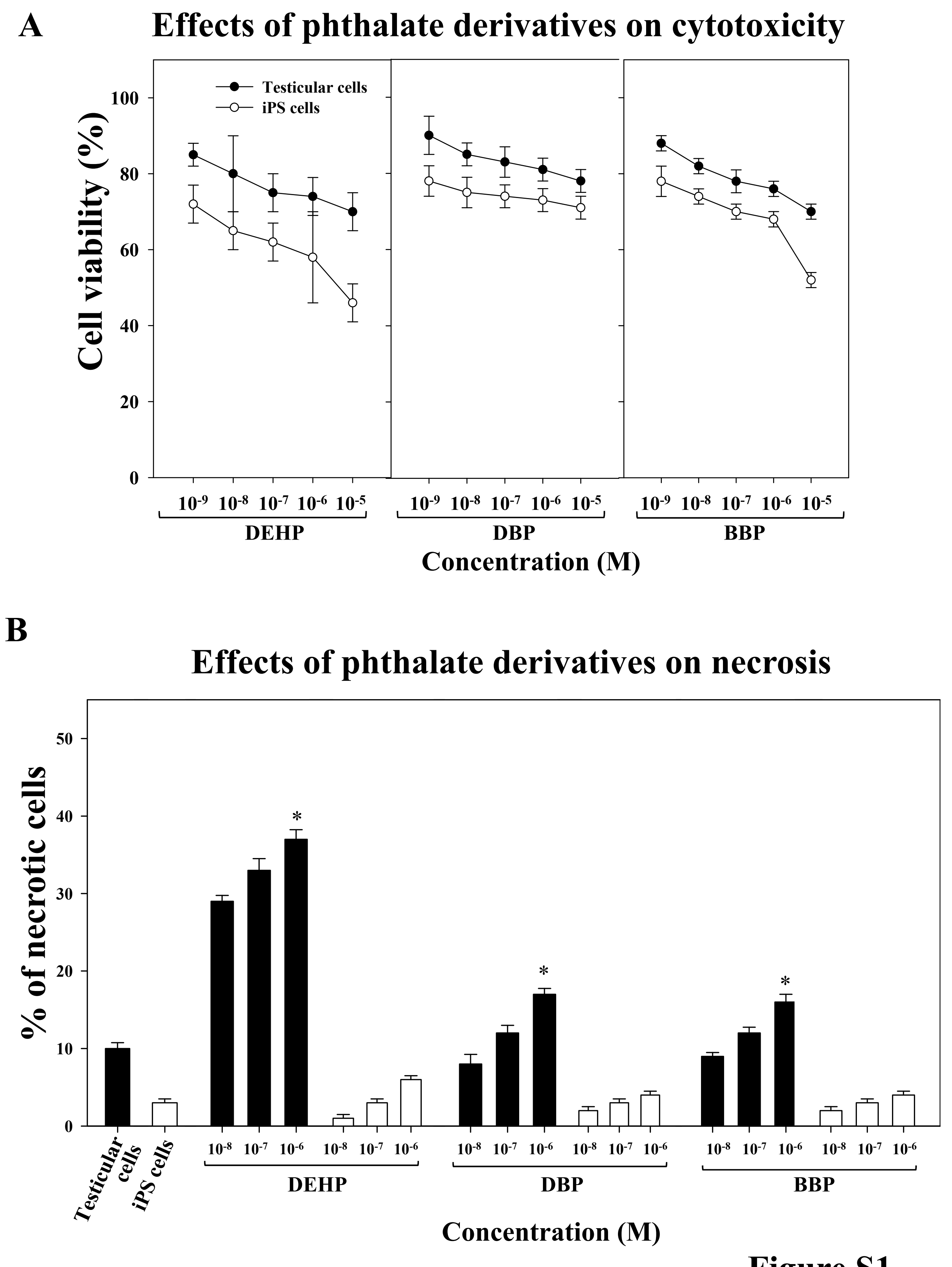
A
Effects of phthalate derivatives on cytotoxicity
Testicular cells
iPS cells
Cell viability (%)
0
20
40
60
80
100
10-9 10-8 10-7 10-6 10-5
DEHP
DBP
BBP
Concentration (M)
B
Effects of phthalate derivatives on necrosis
% of necrotic cells
0
10
20
30
40
50
*
Testicular cells
iPS cells
10-8 10-7 10-6 10-8 10-7 10-6
DEHP
DBP
BBP
Concentration (M)

Figure S1

C

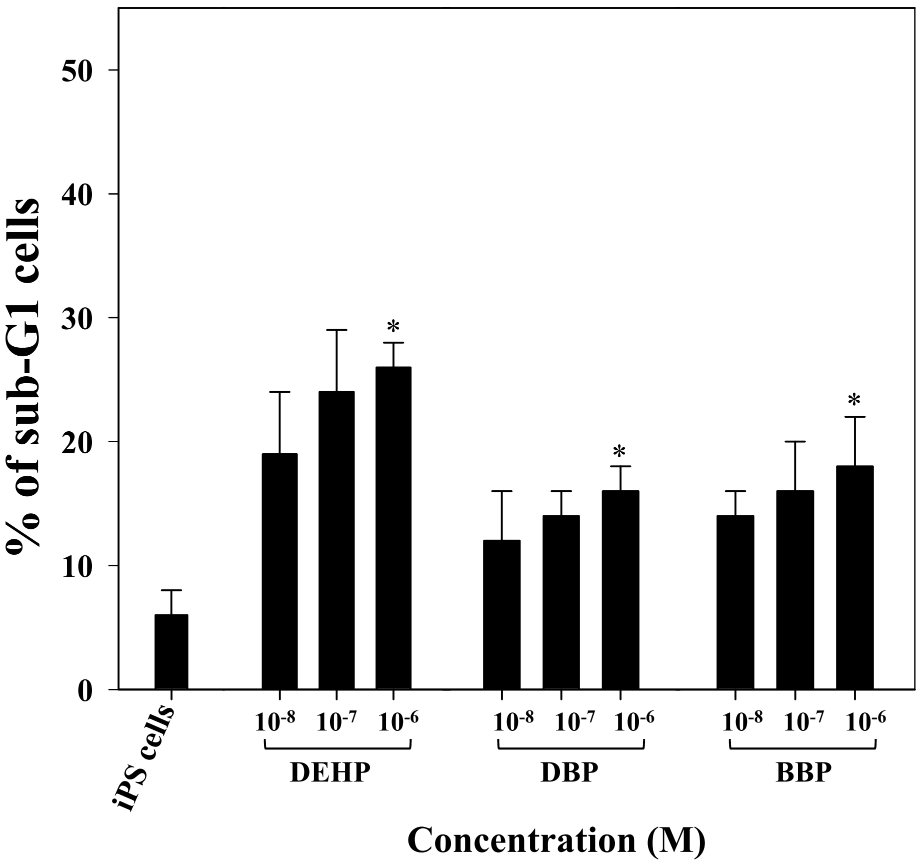
% of sub-G1 cells
0
10
20
30
40
50
*
*
*
iPS cells
10-8 10-7 10-6
DEHP
10-8 10-7 10-6
DBP
10-8 10-7 10-6
BBP
Concentration (M)

Figure S1

A

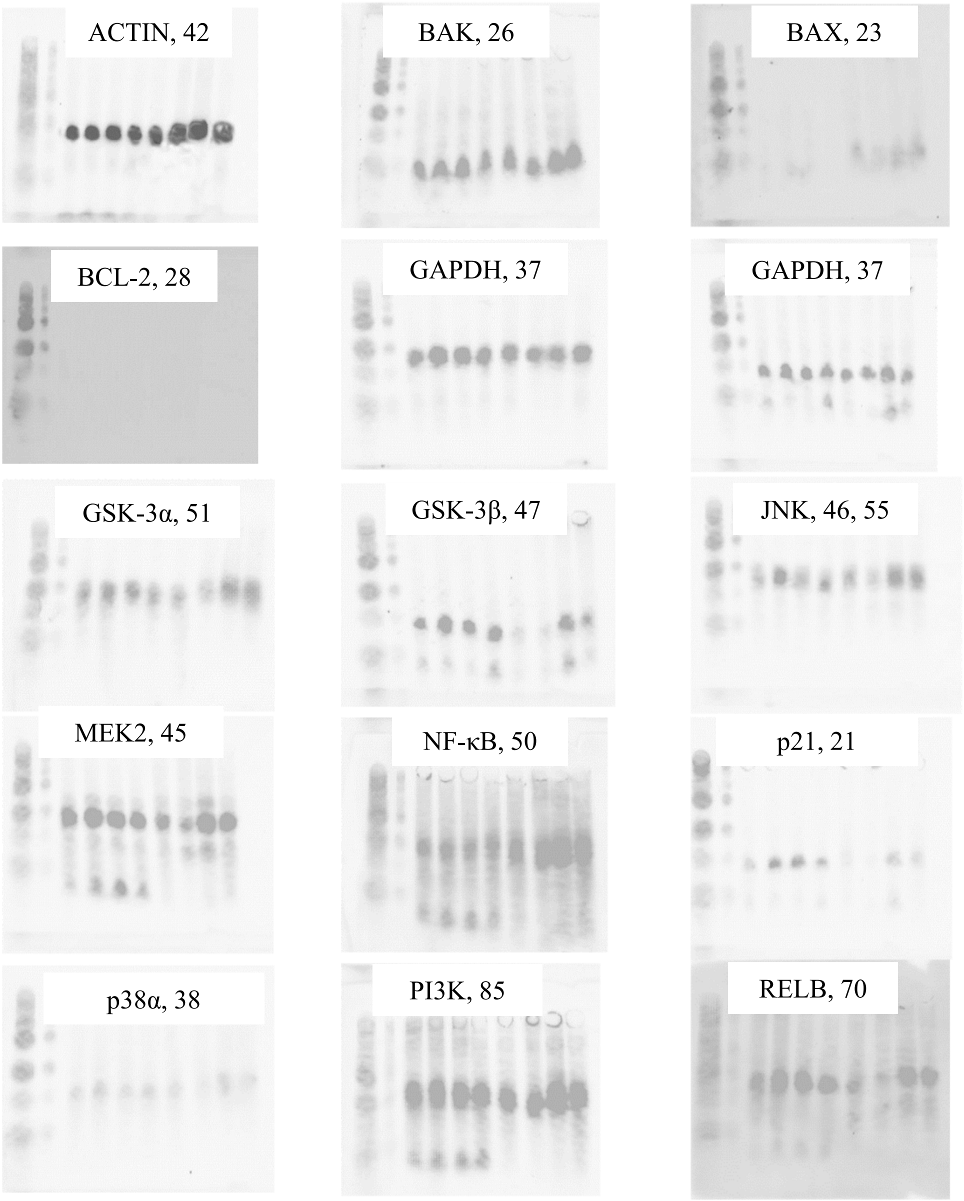
ACTIN, 42
BAK, 26
BAX, 23
BCL-2, 28
GAPDH, 37
GAPDH, 37
GSK-3α, 51
GSK-3β, 47
JNK, 46, 55
MEK2, 45
NF-κB, 50
p21, 21
p38α, 38
PI3K, 85
RELB, 70

Figure S2

B

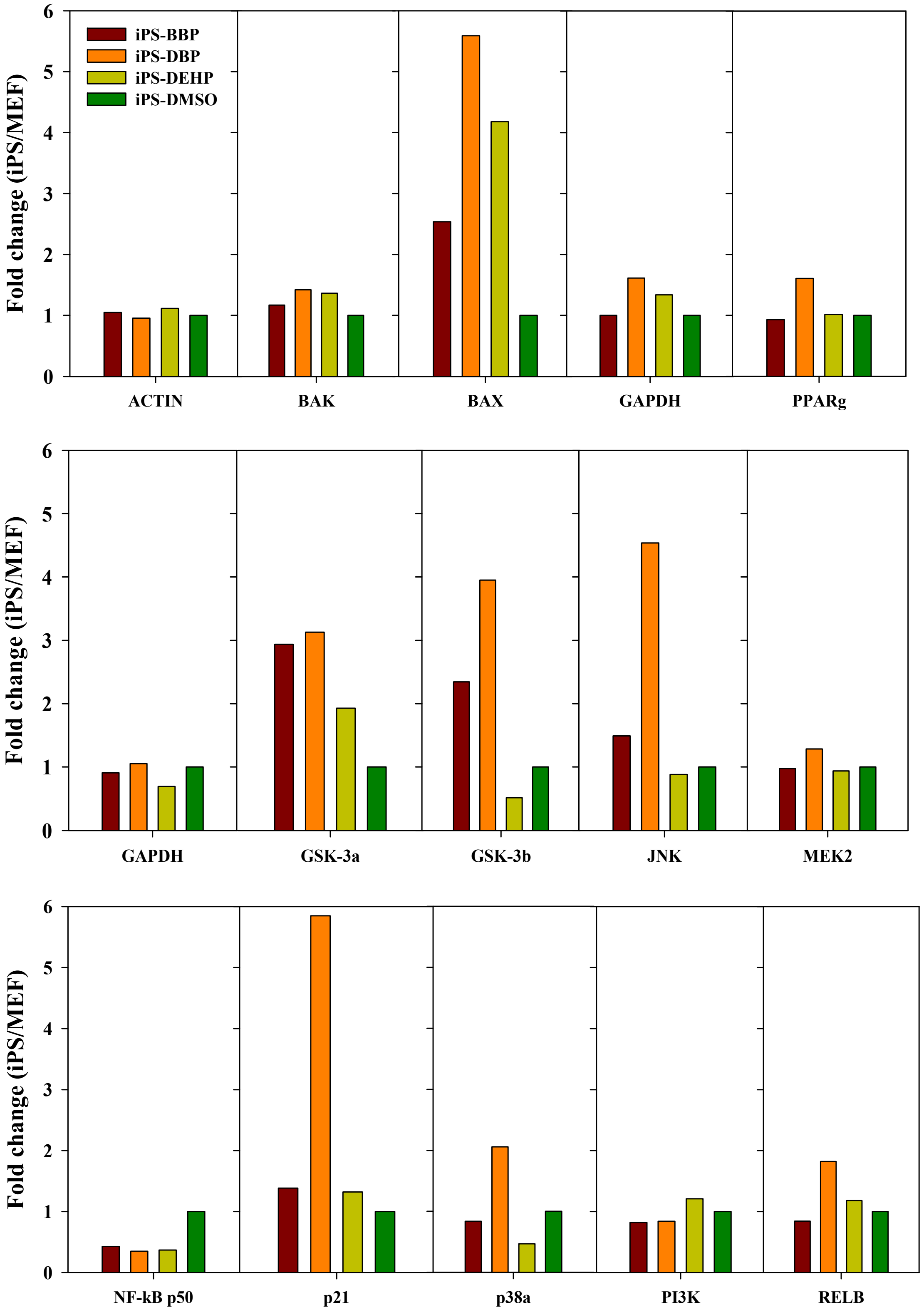
Fold change (iPS/MEF)
iPS-BBP
iPS-DBP
iPS-DEHP
iPS-DMSO
0
1
2
3
4
5
6
ACTIN
BAK
BAX
GAPDH
PPARg
GAPDH
GSK-3a
GSK-3b
JNK
MEK2
NF-kB p50
p21
p38a
PI3K
RELB

Figure S2

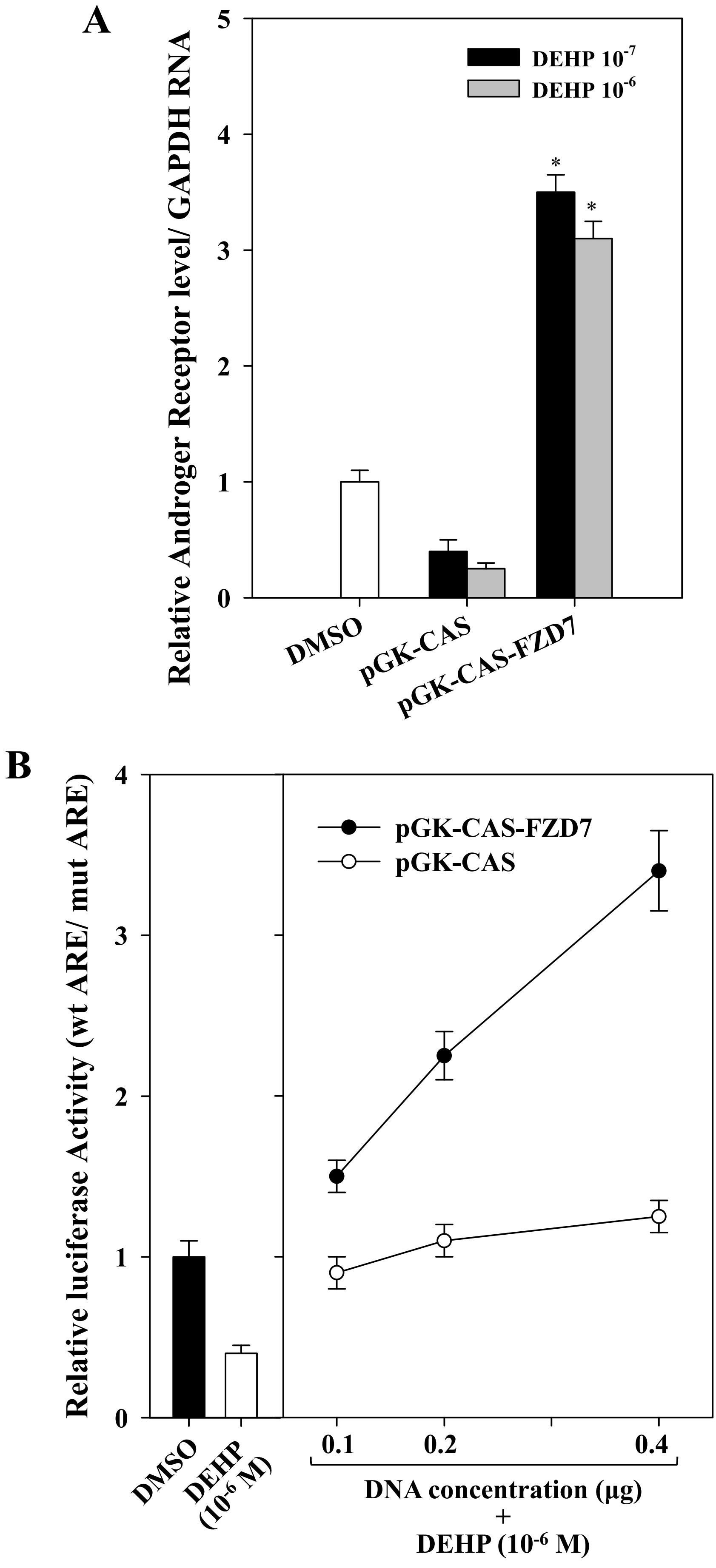
A
B
DEHP 10-7
DEHP 10-6
*
*
Relative Androger Receptor level/ GAPDH RNA
0
1
2
3
4
5
DMSO
pGK-CAS
pGK-CAS-FZD7
pGK-CAS-FZD7
pGK-CAS
Relative luciferase Activity (wt ARE/ mut ARE)
0
1
2
3
4
DMSO
DEHP (10-6 M)
0.1
0.2
0.4
DNA concentration (µg)
+
DEHP (10-6 M)

Figure S3

C

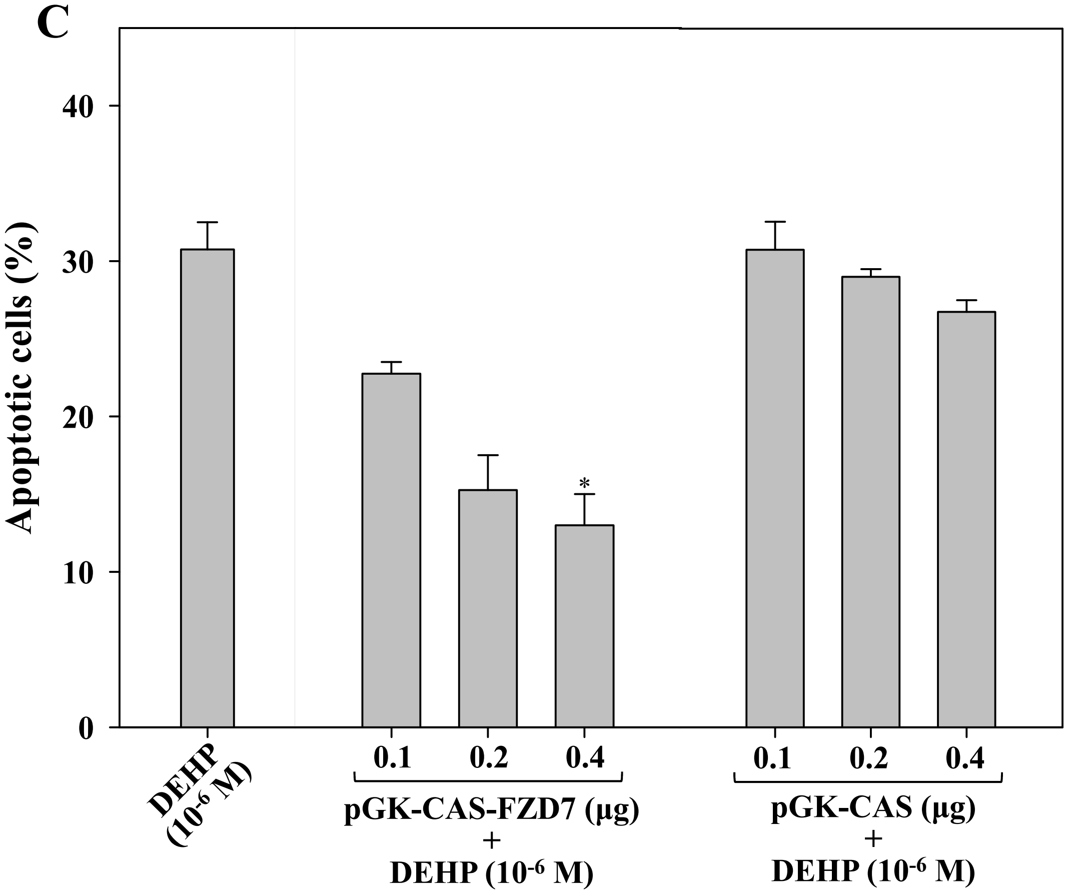

Apoptotic cells (%)
0
10
20
30
40
*
DEHP (10-6 M)
0.1
0.2
0.4
pGK-CAS-FZD7 (µg)
+
DEHP (10-6 M)
0.1
0.2
0.4
pGK-CAS (µg)
+
DEHP (10-6 M)

Figure S3
