# Supplementary material for: Androgen receptor-mediated apoptosis in bovine testicular induced pluripotent stem cells in response to phthalate esters
Source: Cell Death Dis. 2013 Nov 7;4(11):e907–. doi: 10.1038/cddis.2013.420 (PMC3847308; doi:10.1038/cddis.2013.420)
Supplement: Supplementary Information [file cddis2013420x2.doc]

**Supplementary Materials**

Supplementary Information

References

Supplementary Figure legends

SFigure 1

SFigure 2

SFigure 3

**Supplementary Information**

DBP, BBP, and DEHP were purchased from Sigma-Aldrich (St. Louis, MO, USA). Caspase 3 assay kit was from Promega (Madison, WI, USA).　The plasmids *pGK-CAS-FZD7* and *pGK-CAS* were kind gifts of Karl Willert (University of California, San Diego, CA, USA), respectively.

**Cell viability, and necrotic cells.** The number of viable cells was determined using a LIVE/DEAD® Viability/Cytotoxicity Assay Kit (L-3224; Life Technologies, Grand Island, NY, USA). The percentages of necrotic cells were determined using an Apoptotic/Necrotic Cells Detection Kit (PK-CA 707-30017; PromoCell GmbH, Heidelberg, Germany).

**Sub-G1 fraction.** Cells were fixed with 70% ethanol and stained with PI (50 μg/mL) in the presence of RNAse A (100 U/mL). PI-stained cells were detected with the FL-2 photomultiplier of the FACScalibur flow cytometer (BD Biosciences, San Jose, CA, USA). The percentage distribution of cells in the different phases of the cell cycle was determined. The fraction of apoptotic cells was quantified by analysis of the sub-G1 peak (sub-diploid cells).^1^ Three independent experiments were performed for each assay.

**RNA extraction, reverse transcription polymerase chain reaction (RT-PCR) and quantitative PCR (qPCR).** RNA was extracted from cells in the presence of the indicated dose of DEHP, DBP, or BBP and dimethyl sulfoxide (DMSO) as described in Materials and Methods. Real-time quantitative RT-PCR (qPCR) was performed in a PRISM™ 7700 system as described elsewhere (Amersham Biosystems, Foster City, CA, USA). We designed the primers using the public-domain Primer 3 program of GENETYX-Mac Ver. 14 software (Hitachi Software, Tokyo, Japan). The respective pairs of primers are listed in Table II.

**Microwestern arrays.** Cells were lysed at the indicated time points, and Microwestern arrays (MWA) were conducted to measure protein expression and modification as previously described.^2^The intensity of bands in Western blotting was quantitated by GeneTools (SYNGENE, Cambridge, UK) and Image Lab^TM^ software (BIO-RAD, Hercules, CA, USA). The relative values of each band-image in iPSCs were calculated by normalization of corresponding band image of MEFs as 1.0.

**Transfection and luciferase assay.** *pIRESneo-AR, pIREneo, p21-Luc, p21/dlMscI, p3PREc-Luc, pE1B-Luc* were transfected into bovine iPSCs and MEFs at 400 ng of total DNA per well of a 24-well plate (5 × 10^4^ cells/well) using 2 µL of lipofectamine^TM^-2000 reagent (Invitrogen) and cultured in the presence of the indicated amount of phthalate ester, and luciferase activity was measured using an assay kit system (Dual-Glo; Promega) as described in Materials and Methods. Twenty-four hours after phthalate treatment, luciferase activity was measured using the commercial luciferase assay system (Dual-Glo). Relative luciferase activity is expressed as the ratio of the luciferase activities in iPSCs and MEFs. Control activities are derived from the cells treated with DMSO.

**References**

1. Ormerod MG, Collins MK, Rodriguez-Tarduchy G, Robertson D. [Apoptosis in interleukin-3-dependent haemopoietic cells. Quantification by two flow cytometric methods.](http://www.ncbi.nlm.nih.gov/pubmed/1517601) *J Immunol Methods* 1992; **53**: 57-65.

2. Ciaccio MF, Wagner JP, Chuu CP, Lauffenburger DA, Jones RB. System analysis of EGF receptor signaling dynamics with microwestern arrays. *Nat Method* 2009; **7**: 148-155.

3. Pan J, Nakade K, Huang YC, Zhu ZW, Masuzaki S, Hasegawa H, Murata T, Yoshiki A, Yamaguchi N, Lee CH, Yang WC, Tsai EM, Obata Y, Yokoyama KK. [Suppression of cell-cycle progression by Jun dimerization protein-2 (JDP2) involves downregulation of cyclin-A2.](http://www.ncbi.nlm.nih.gov/pubmed/20802531) *Oncogene* 2010; **29**: 6245-6256.

4. Jin C, Kato K, Chimura T, Yamasaki T, Nakade K, Murata T, Li H, Pan J, Zhao M, Sun K, Chiu R, Ito T, Nagata K, Horikoshi M, Yokoyama KK. Regulation of histone acetylation and nucleosome assembly by transcription factor JDP2. *Nat Struc Mol Biol* 2006; **3**: 331-338.

**Figure legends**

**Supplementary Figure S1.** Phthalate esters induced cytotoxicity, necrosis and apoptosis. (**A**)

Cell viability was measured by a LIVE/DEAD® Viability/Cytotoxicity Assay Kit (L-3224; Life Technologies, Grand Island, NY, USA) in the presence or absence of phthalate esters for 48 h. (**B**) The percentages of necrotic cells were determined using an Apoptotic/Necrotic Cells Detection Kit (PK-CA 707-30017; PromoCell GmbH, Heidelberg, Germany) by incubation of cells with various concentrations of phthalate derivatives for 48 h. The data were expressed as the means ± S.D. and a *t*-test was used to compare them with the results obtained with DMSO-treated control testicular cells (n ≥ 3, **P* < 0.05). (**C**) The sub-G1 fraction of cells stained with PI (50 μg/mL) was determined by FACS analysis as described in Materials & methods. The data were expressed as the means ± S.D. and a *t*-test was used to compare them with the results obtained with DMSO-treated control iPSCs (n ≥ 3, **P* < 0.05).

**Supplementary Figure S2.** Representative patterns and quantitation of MWA. The protein samples from bovine iPSCs with MEF feeder cells and MEF feeder cells only were prepared and run in MWA format.^2^ (**A**) One of the typical patterns of MWA are shown. MEFs were treated with mitomycin C, cultured in the iPSC medium for 2 weeks, and treated with the phthalates indicated [0.1% dimethyl sulfoxide (DMSO)-treated control, 10^–6^ M di-(2-ethylhexyl) phthalate (DEHP), 10^–6^ M di (*n*-butyl) phthalate (DBP), and 10^–6^ M butyl benzyl phthalate (BBP)] for 24 h, as described in the Materials and Methods, and then harvested. Lysates from iPSCs and MEFs along with protein ladder were applied to the gel and blotted, and stained with the appropriate antibodies against apoptosis- and cell cycle- related proteins. (**B**) Relative expression values of the blotted proteins in iPSCs and MEF feeder cells. Blots were scanned and quantified using a LI-COR Odyssey near-infrared imaging system. β-Actin and glyceraldehyde-3-phosphate dehydrogenase (Millipore) were used as loading controls. The intensity of bands in Western blotting was quantitated by GeneTools (SYNGENE, Cambridge, UK) and Image Lab^TM^ software (BIO-RAD, Hercules, CA, USA). The relative intensities of each band-image in iPSCs were calculated by normalization of corresponding band-image of MEFs as 1.0.

**Supplementary Figure S3.** Effect of forced expression of *FZD7* on *AR* gene expression and apoptosis in iPSCs. (**A**) Four hundred nanograms of *pGK-CAS-FZD7* and control *pGK-CAS* were introduced into bovine iPSCs treated with 10^-6^ or 10^-7^ M DEHP, culture for 24 h. The relative expression of *AR* was calculated by qPCR as described Materials and Methods. The data are presented as means ± SD and statistically analyzed with *t*-test, compared with control DMSO treated iPSCs; n ≥ 3, **P* < 0.05. (**B**) Effect of DNA concentration of *pPGK-CAS-FZD7*and *pGK-CAS* on AR promoter-luciferase activity (100-400 ng) in bovine iPSCs treated with 10^-6^ M DEHP as described in Materials and Methods. (**C**) Effect of FZD7 expression on apoptosis. Apoptotic cells were quantified using Apoptotic/Necrotic Cells Detection Lit (PK-CA 707-30017; PromoCell GmbH). The data were expressed as the means ± S.D. and a *t*-test was used to compare them with the results obtained with DMSO-treated control iPSCs (n ≥ 3, **P* < 0.05).
